# “Oh, Dear We Are in Tribble”: An Overview of the Oncogenic Functions of Tribbles 1

**DOI:** 10.3390/cancers16101889

**Published:** 2024-05-16

**Authors:** Karnika Singh, Christian A. Showalter, Heather R. Manring, Saikh Jaharul Haque, Arnab Chakravarti

**Affiliations:** Department of Radiation Oncology, The Ohio State University Comprehensive Cancer Center, Columbus, OH 43210, USA

**Keywords:** tribbles 1, pseudokinase, treatment resistance, oncogenic signaling, cancer, lipid metabolism, drug targeting

## Abstract

**Simple Summary:**

Pseudokinases have evolved from conventional kinases and differ from them in their inability to phosphorylate their substrates. The Tribbles pseudokinases have evolved from the CAMK family of kinases and include three members: Tribbles 1, 2, and 3. Tribbles 1, which evolved later in metazoan lineage, appears to have a regulatory function. It interacts with a variety of substrates through different domains embedded in its molecular structure. Each interaction regulates cellular processes involved in cell division, survival, and metabolism. These processes also extrapolate to cancer development and other diseases. Based on its involvement in disorders and therapy resistance, it can be considered a potential candidate for drug development. The improved knowledge about its function can be utilized to design small-molecule inhibitors against TRIB1.

**Abstract:**

Pseudokinases are catalytically inactive proteins in the human genome that lack the ability to transfer phosphate from ATP to their substrates. The Tribbles family of pseudokinases contains three members: Tribbles 1, 2, and 3. Tribbles 1 has recently gained importance because of its involvement in various diseases, including cancer. It acts as a scaffolding protein that brings about the degradation of its substrate proteins, such as C/EBPα/β, MLXIPL, and RAR/RXRα, among others, via the ubiquitin proteasome system. It also serves as an adapter protein, which sequesters different protein molecules and activates their downstream signaling, leading to processes, such as cell survival, cell proliferation, and lipid metabolism. It has been implicated in cancers such as AML, prostate cancer, breast cancer, CRC, HCC, and glioma, where it activates oncogenic signaling pathways such as PI3K-AKT and MAPK and inhibits the anti-tumor function of p53. TRIB1 also causes treatment resistance in cancers such as NSCLC, breast cancer, glioma, and promyelocytic leukemia. All these effects make TRIB1 a potential drug target. However, the lack of a catalytic domain renders TRIB1 “undruggable”, but knowledge about its structure, conformational changes during substrate binding, and substrate binding sites provides an opportunity to design small-molecule inhibitors against specific TRIB1 interactions.

## 1. Introduction

The human genome encodes a total of 581 protein kinases, of which 50 are catalytically inactive or weak and termed pseudokinases because they lack at least one residue in their conserved catalytic site [1]. Some pseudokinases retaining weak kinase activity include ErbB3/HER3, in which a couple of amino acids in its catalytic domain are replaced by other amino acids (Glu740> His and Asp815> Asn), but it is still able to bind ATP and catalyze phosphoryl transfer with lower efficiency [2]. Pseudokinases are known to perform a variety of physiological functions, such as allosteric regulation of other enzymes (e.g., VRK3 [3]), scaffolding of signaling components (e.g., Tribbles family [4]), and acting as molecular switches (e.g., MLKL [5]). Many pseudokinases are implicated in several diseases, such as cancer, metabolic disorders, and autoimmune diseases, among others [6]. One such pseudokinase is mammalian tribbles homolog 1 (TRIB1), a member of the Tribbles family of pseudokinases, which has emerged to play a role in several diseases, including cancer, and is the focus of this review.

## 2. Structure and Function of TRIB1

TRIB1 is encoded by the *TRIB1* gene on chromosome 8 (8q24.13 locus) in the human genome. It is also known as C8FW, TRB1, GIG2, and SKIP1. TRIB1 was first identified in 1997 by Wilkin and colleagues (human clone C8FW), and in spite of a ~30% sequence identity with the protein kinase family, they did not observe an ATP binding site in their isolated clone [7]. The half-life of *TRIB1* mRNA is less than 1 h [8], and protein turnaround is about 2 h [9]. The TRIB1 protein has two isoforms, isoforms 1 and 2. The isoform 1 encoded by the longer transcript is the full-length protein (41 kDa), whereas the translation of isoform 2 is initiated at a downstream start codon, and the encoded protein contains a shorter N-terminus (23.48 kDa) [10]. *TRIB1* is highly expressed in the bone marrow, liver, thyroid gland, urinary bladder, adipose tissue, gall bladder, and parathyroid gland [11]. TRIB1 protein is found in both the cytoplasmic [12] and nuclear [13] compartments of the cell. Several transcription factor binding sites are predicted to be present in the *TRIB1* gene promoter 5′ UTR region, including E2F, C/EBPβ, and NF-1 [4]. Similarly, many miRNAs also putatively bind to the 3′UTR of *TRIB1* mRNA and regulate its gene expression and function [14]. ATF3 regulates *TRIB1* gene expression in response to proteasome inhibition in hepatocyte cell lines [15]. 

### 2.1. Evolution and Structure of TRIB1

TRIB1 belongs to the Tribbles family of pseudokinases, comprised of TRIB1, TRIB2, TRIB3, and a distantly related member, STK40. The name Tribbles comes from their homology with the domain structure of the *Drosophila* tribbles protein [16]. In 2000, TRIB1 was first described to have a function during *Drosophila* gastrulation in the modulation of String/Cdc25 proteins in mesoderm cells [16,17,18]. TRIB1 orthologs are mainly encountered in later metazoan lineages (the vertebrates), as opposed to TRIB2, which is also observed in the oldest metazoans [4]. This is suggestive of the fact that TRIB1 evolved later in evolution, possibly to accommodate complex cell signaling processes in higher organisms. 

The structure of TRIB1 is composed of three domains: The N-terminal domain (AA 1–90), the central pseudokinase domain (AA 91–330), and the C-terminal domain (AA 331–373) [19,20]. The N-terminal domain of TRIB1 contains a putative nuclear localization signal spanning residues 33–51, which is shown to be required for nuclear localization [9]. The N-terminal domain is also rich in proline (P), glutamate (E), serine (S), and threonine (T) residues (AA 53–88), which are characteristic of PEST domains; however, it is also shown to be unassociated with TRIB1 instability [15]. The pseudokinase domains of TRIB1, 2, and 3 share sequence similarity with the canonical kinase domain of CAMK (calcium/calmodulin-dependent protein kinase) [21]. Furthermore, at the amino acid level, TRIB1 shares 71% sequence similarity with TRIB2 and 54% sequence similarity with TRIB3 in its pseudokinase domain region. The structural features of the pseudokinase domain that distinguish it from a canonical kinase include a bent αC helix and divergent glycine-rich loop, both of which are required for ATP binding in a bona fide protein kinase. The Asp-Phe-Gly (DFG) motif is replaced by the Ser-Leu-Glu (SLE) sequence that occludes the nucleotide binding pocket. All of these structural/sequence changes represent a deformed N-terminal lobe and prevent the binding of ATP to TRIB1, thus lacking or inhibiting any phosphoryl transfer, which in turn classifies it as a pseudokinase [22]. The pseudokinase domain also allows for binding of interacting partners and substrates, with C/EBPα/β (AA 168–293 [23]) being its most characterized substrate so far. The C-terminal domain of TRIB1 houses two important protein–protein interaction motifs: ILLHPWF (AA 332–339 [12]) that binds MEK1/2, and DQIVPE (AA 355–360 [24]) for binding COP1 E3 ubiquitin ligase. It has been shown by Murphy et al. that there is an intramolecular interaction between the C-terminal tail of TRIB1 and its pseudokinase domain, which in turn increases the stability of the TRIB1 protein [22]. This interaction is also observed in the CAMK group of protein kinases, where the C-terminal regulatory region makes multiple inhibitory interactions with its catalytic core to play an autoinhibitory role [25]. Both TRIB2 and TRIB3 also contain a MEK1/2 binding site, I(L/D)LHPW(F/L) (AA 30(2/9)-3(07/15)), and a COP1 binding site (D/A)Q(L/V)VPD (AA 3(26/33)-3(31/37)) on their C-termini [26]. Overall, TRIB2 (343 amino acids) and TRIB3 (358 amino acids) are smaller proteins than TRIB1, with a shorter N-terminal domain as revealed by amino acid alignment.

### 2.2. Functions of TRIB1

TRIB1 has been recognized as an adapter protein that provides a scaffold for the degradation of its substrates (summarized in Table 1). The most characterized substrate of TRIB1 is C/EBPα, a lineage-specific transcription factor that undergoes degradation via COP1 E3 ubiquitin ligase [22]. Binding of C/EBPα to the TRIB1 pseudokinase domain initiates a conformational change in the SLE motif to release the COP1 binding motif on the C-terminal domain, in turn relieving the autoinhibition. This substrate-bound conformation of TRIB1 is known as the “SLE-in” conformation and corresponds to the active protein kinase conformation [23]. COP1 then binds to the C-terminus through its WD40 domain [24] and causes ubiquitination of C/EBPα at its lysine residues, ultimately causing its degradation through the ubiquitin proteasome system [27]. After the degradation of C/EBPα, the C-terminal domain folds itself back onto the pseudokinase domain, and the autoinhibitory conformation is restored. This conformation is known as “SLE-out” and is similar to an inactive protein kinase [28]. The tribbles protein also controls the protein levels of *slbo*, the *Drosophila* homolog of the C/EBP family of transcription factors, via degradation and inhibits border cell migration during oogenesis [29]. Furthermore, because the C/EBP family of transcription factors determines the switch between granulopoiesis and monopoiesis [30], degradation of C/EBPα by TRIB1 regulates myeloid cell differentiation [31]. TRIB1 has also been shown to negatively regulate retinoic acid receptor (RAR) signaling by forming a complex with it and its binding partner RXR (retinoid X receptor) [32].

The other primary function of TRIB1 is the sequestration of signaling molecules and subsequent activation of the downstream pathways (summarized in Table 1). As mentioned above, the pseudokinase and the C-terminal domains of TRIB1 contain sites that bind to Akt and MEK1/2, respectively. *Drosophila* tribbles have been shown to interact with Akt1 through its kinase-like domain and inhibit its activation [33]. Another report shows that TRIB1 binds to Akt, possibly at its pseudokinase domain, and causes its activation [34]. TRIB3 has also been shown to interact with Akt1 through its pseudokinase domain [35]. The mediation of Akt activation and its downstream signaling by TRIB1 suggests that TRIB1 is a modulator of the PI3K-Akt pathway [36]. Alternatively, TRIB1 also interacts with MEK1/2 through its C-terminal domain and regulates the activation of its downstream protein, ERK [37]. TRIB1 also interacts with MKK4 through its pseudokinase domain and modulates vascular smooth muscle cell proliferation and chemotaxis by activating the downstream JNK pathway [38]. TRIB1 also plays a role during M1 and M2 macrophage polarization of murine bone marrow-derived macrophages (BMDMs) by modulating the JAK/STAT pathway [39]. TRIB1 has been shown to be important for the differentiation of tissue-resident M2-like macrophages (F4/80^+^ MR^+^), which are involved in the maintenance of adipose tissues [31].

**Table 1:** The interacting partners of TRIB1.

Interacting Partner	Interaction Site	Function/Effect	References
C/EBPα/β	Pseudokinase domain (AA168–293)	Degradation of C/EBPα/β	[22,23]
COP1	C-terminal domain (AA 355–360)	Proteasome-mediated degradation of substrates such as C/EBPα/β	[22,24]
MEK1/2	C-terminal domain (AA 332–339)	MAPK pathway activation	[12,37]
RAR/RXRα	Pseudokinase domain	Downregulation of RAR signaling	[32]
Akt	Pseudokinase domain (AA 90–160)	Cell survival through the activation of PI3K-Akt pathway	[34,36]
MKK4	Pseudokinase domain	JNK pathway activation, vascular smooth muscle cell proliferation and chemotaxis	[38]
MLXIPL (ChREBP)	Pseudokinase domain	Degradation of MLXIPL (ChREBP) leading to transcriptional inhibition of lipogenesis genes	[40]
HNF4A	Pseudokinase domain, multiple epitopes but AA 1–240 exhibit strongest binding	Gene expression of lipid metabolism and lipoprotein regulators and genes in liver and intestine	[41]
SAP18/Sin3A	COP1 binding site	Modulation of MTTP expression involved in lipid metabolism	[42]
Nrf2	C-terminal domain	Nrf2 sequestration in cytoplasm suppressing liver regeneration	[43]
NF-кB	N-terminal and Pseudokinase domain	Direct promoter recruitment to NF-кB DNA recognition site and upregulation of cytokine gene expression	[44]
FoxP3	N-terminal and Pseudokinase domain	Tregs regulation	[45]
FERMT2	Pseudokinase domain	Degradation of FERMT2 and transcriptional regulation through β-catenin	[46]
ZBT7A	Unknown	Modulation of ER-associated transcription	[46]
CUL4A/B	C-terminal domain	Degradation of substrates	[46]
HDAC1	Unknown	P53 deacetylation	[47]
CD72	Pseudokinase domain	Degradation of CD72 leading to development of autoimmunity	[48]
MALT1	N-terminal domain (AA 83–89)	T cell receptor signaling regulation	[49]

#### 2.2.1. TRIB1 and Lipid Metabolism

TRIB1 is known to play an important role in lipid metabolism. It was first identified to be associated with triglycerides, LDL, and HDL cholesterol in a genome-wide association study (GWAS) [50]. *TRIB1* knockout mice exhibit increased triglyceride and plasma cholesterol levels, which are accompanied by downregulation of lipogenic genes such as acetyl-CoA carboxylase and fatty acid synthase, among others [51]. Liver-specific deletion of *TRIB1* has revealed that elevated levels of C/EBPα increase the transcription of fatty acid synthesis genes and result in pathologic phenotypes, suggesting a role of TRIB1 in keeping the transcription of lipogenic genes in check via degradation of C/EBPα [52,53]. However, on the contrary, a recent report suggests that adipocyte specific knockdown of *TRIB1* results in reduced plasma triglyceride and cholesterol levels along with increased adiponectin secretion, which are associated with improved metabolic health in humans [54]. TRIB1 also interacts with the hepatic lipogenic master regulator MLXIPL (MLX interacting protein like), also known as ChREBP, and causes its degradation, leading to transcriptional inhibition of genes involved in liponeogenesis [40]. The physical interaction of TRIB1 with HNF4A has also been reported, which plays a role in liver and intestinal gene expression as well as lipid metabolism [41]. TRIB1 also acts as a scaffold for the SAP18 (Sin3A-associated protein) and mSin3A interactions, which modulate the expression of *MTTP* (microsomal TG transfer protein) in the mouse liver, ultimately impacting lipid metabolism [42]. TRIB1 also has the capability to suppress liver regeneration by modulating redox homeostasis in hepatocytes via blockade of Nrf2 nuclear translocation [43]. *TRIB1* genetic loci are also associated with liver function in terms of certain enzymes’ secretion, such as alanine transaminase (ALT), alkaline phosphatase (ALP), and γ-glutamyl transferase (GGT), which are commonly used as markers of certain liver ailments, suggesting it to be a genetic trait that might be inherited among humans [55]. 

#### 2.2.2. TRIB1 and Innate Immunity

**Figure 1:**
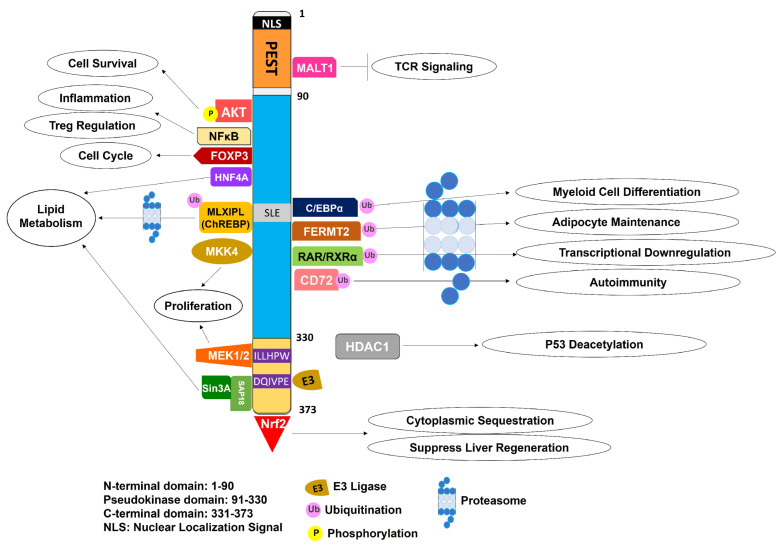
Overview of TRIB1 structure and functions. TRIB1 has three domains: An N-terminal domain, a pseudokinase domain, and a C-terminal domain. All three domains bind to distinct substrates and interacting proteins to carry out different functions. The N-terminal domain contains a NLS (nuclear localization signal/sequence) and a typical PEST domain. It is shown to bind MALT1 and regulate TCR (T cell receptor) signaling. The pseudokinase domain is the longest domain and acts as a scaffold and sequester a variety of proteins. It is known to bind NF-кB, Foxp3, HDAC1, HNF4A, Akt, and MKK4 and influence processes such as inflammation, cell cycle regulation, p53 deacetylation, lipid metabolism, Treg regulation, cell survival, and proliferation. The pseudokinase domain also binds substrates for proteasomal degradation such as C/EBPα/β, MLXIPL (ChREBP), FERMT2, RAR/RXRα, and CD72. Degradation of these substrates affects processes such as myeloid cell differentiation, lipid metabolism, adipocyte maintenance, transcriptional downregulation, and autoimmunity. The pseudokinase domain also contains an SLE sequence as a substitute for the DFG motif that is responsible for binding ATP in a canonical kinase. The C-terminal domain binds MEK1/2 at ILLHPW sequence and activates downstream MAPK signaling to induce cell proliferation. COP1 E3 ligase also binds at the DQIVPE sequence in the C-terminal domain to result in degradation of TRIB1 substrates through the ubiquitin proteasome system. CUL4A/B is another ubiquitin ligase that is shown to bind TRIB1. Nrf2 also binds to TRIB1 through the C-terminal domain, and this interaction has been implicated in liver regeneration.

TRIB1 regulates NF-кB through various mechanisms, namely, regulation of p100 expression and inhibition of IKKα via phosphorylation by Akt on T23, among others. Regulation of NF-кB by TRIB1 is supported by *TRIB1* knockdown studies, which resulted in downregulation of NF-кB-transcribed genes such as *CCND1* (cyclin D1) and *IL-8* (interleukin 8), suggesting a role of TRIB1 in cell cycle transition and inflammation [36]. Furthermore, the IL-8 promoter contains AP-1 and C/EBPα binding sites, both of which have been shown to be regulated by TRIB1 [19]. TRIB1 is also shown to interact with the RelA subunit of NF-кB in white adipocytes and acts as its transcriptional co-activator to regulate client cytokine gene expression [44]. Interaction of TRIB1 with FoxP3, the master regulator of regulatory T cells (Tregs), is reported in human adherent cell lines and Tregs [45,56]. TRIB1 also increases IL-2 production in activated T cells through NFAT (nuclear factor of activated T cells) [57]. TRIB1-mediated downregulation of C/EBPβ results in altered TLR-mediated signaling in macrophages, especially in the context of C/EBPβ-induced gene expression [58]. Knockdown of *TRIB1* in macrophages inhibits their migration and increases production of TNFα [59]. TRIB1 is identified as a potential biomarker for chronic antibody-mediated allograft rejection due to its increased mRNA levels present in peripheral blood mononuclear cells of patients with deteriorating renal graft function [60]. Taken together, these reports suggest that TRIB1 may have a function in innate immunity as well. The functional overview of TRIB1 is outlined in Figure 1.

## 3. Role of TRIB1 in Disease

Based on its various functions described above, TRIB1 has been implicated in numerous diseases and malignancies. Its close proximity to *MYC* and its involvement in fundamental pathways such as MAPK signaling, NF-кB signaling, and the PI3K-Akt pathway have led to the identification of TRIB1 as an oncogene that plays a pivotal role in tumor progression and maintenance. Furthermore, its involvement in lipid metabolism and innate immunity causes other disease modalities such as atherosclerosis and inflammation.

### 3.1. TRIB1 and Cancer

#### 3.1.1. Acute Myeloid Leukemia (AML)

It was observed in an AML patient that *TRIB1* was overexpressed along with *MYC,* both of which reside on the same chromosomal region (8q24) 2.25 Mb apart [61]. AML is characterized by the accumulation of hematopoietic progenitor cells in the bloodstream due to genetic alterations affecting their differentiation [62]. As mentioned earlier, C/EBPα is the lineage-specific transcription factor that is targeted for degradation by TRIB1 [22,30]. It was later discovered that overexpressed TRIB1 drives Hoxa9-induced leukemogenesis by decreasing C/EBPα protein levels, thereby modulating the enhancer programs at the *Erg* and *Spns2* loci [63]. Hoxa9 and Meis1 are normally expressed in early progenitor cells and decrease in expression upon their terminal differentiation [64]. In the context of AML, Hoxa9 and Meis1 are overexpressed in primary bone marrow cells and cause leukemogenesis [65,66,67]. Hoxa9 is a regulator of the primitive hemopoietic compartment, and Meis1 is a transcription factor critical for leukemia stem cell regulation [68]. Specific interaction between TRIB1 and the coactivation of Hoxa9/Meis1 is reported in AML [65]. TRIB1-mediated activation of the MAPK pathway also plays a role in AML by enhancing the self-renewal of malignant bone marrow cells [12]. 

#### 3.1.2. Prostate Cancer

*TRIB1* and *cMYC* are observed to be co-amplified in prostate cancer as well. *TRIB1* mRNA levels are also similarly elevated in the prostate of *PTEN* null mice, exhibiting increased proliferation potential [69]. *TRIB1* overexpression causes increased sphere formation in prostate cancer cell lines and increased tumor formation in a xenograft mouse model [70]. TRIB1 influences the prostate cancer tumor microenvironment by causing CD136+ macrophage infiltration, promoting M2 macrophage differentiation, and affecting cytokine secretion by inhibiting IкB-ζ (NF-кB inhibitor zeta) [71].

#### 3.1.3. Breast Cancer

*TRIB1* and *cMYC* co-amplification is further observed in breast cancer patients [46] and associated with reduced overall and disease-free survival [46]. TRIB1 regulates both the G1/S [36] and G2/M transition [46] in breast cancer cells. A recent study utilizing the qPLEX-RIME technique [72] has revealed that TRIB1 interacts with the β-catenin co-factor FERMT2 (FERM Domain Containing Kindlin 2) and ER co-factor ZBTB7A (Zinc Finger and BTB Domain Containing 7A) in breast cancer cells, giving further insights into the TRIB1 interactome and its involvement in transcriptional regulation through β-catenin and ER [46]. TRIB1 reduces DR5 protein levels in breast cancer cells through its elevated NF-кB signaling, thus decreasing TRAIL-induced apoptosis [36]. Heightened expression of TRIB1 in tumor associated macrophages (TAMs) influences the breast cancer tumor microenvironment by regulating oncogenic cytokine expression [73]. 

#### 3.1.4. Colorectal Cancer (CRC)

*TRIB1* has been shown to be amplified in both CRC cell lines and patients [74]. *TRIB1* and *MYC* are co-amplified in CRC cells. TRIB1 protein expression significantly correlates with the expression of proteins such as p-ERK, p-MEK, Akt, PTEN, cleaved caspase 3, and MYC in CRC cells [75]. TRIB1 overexpression correlates with poor overall survival in CRC patients. TRIB1 promotes migration and invasion of CRC cells through the activation of FAK/Src and ERK pathways, resulting in an upregulation of MMP-2 expression [76].

#### 3.1.5. Other Cancers

The *TRIB1* gene is also associated with pancreatic cancer in a Chinese Han population [77]. *TRIB1* is observed to be highly expressed in bone marrow mononuclear cells of multiple myeloma (MM) patients with progressive disease compared to those in remission. Such patients have an increased percentage of M2 macrophages, suggesting that TRIB1 plays a role in M2 macrophage polarization in MM [78]. TRIB1 plays a role in hepatocellular carcinoma (HCC) as well, as evidenced by its upregulation in HCC cell lines and tumor tissues. TRIB1 promotes cell proliferation, migration, invasion, and epithelial-to-mesenchymal transition (EMT) in HCC cell lines. TRIB1 upregulation is accompanied by p53 downregulation and increased β-catenin signaling [79], as observed in breast cancer cells. A previous report suggests that TRIB1 can downregulate p53’s transcriptional activity by forming a complex with HDAC1, leading to HDAC1 catalyzed p53 deacetylation, thereby reducing its DNA binding activity [47]. Moreover, it has been shown in glioma and primary GBM cells that TRIB1 reduces p53 levels through a similar mechanism [34,80]. Increased *TRIB1* mRNA levels have been correlated with poor overall survival of glioma patients [34]. This mechanism suggests that TRIB1 could play a central role in tumor initiation and maintenance by modulating the tumor suppressor activities of p53. 

### 3.2. TRIB1 and Other Diseases

In addition to cancer, TRIB1 has been implicated in metabolic and lipid disorders, according to several GWAS. *TRIB1* variants are directly associated with a reduced risk of myocardial infarction in humans [51]. Similarly, a previous review has discussed that TRIB1 is linked to the etiology of Crohn’s disease, non-alcoholic fatty liver disease, dyslipidemia, and cardiovascular disease [81]. Increased *TRIB1* gene expression is encountered in the coronary arteries of patients with ischemic heart disease [38]. Genetic deletion of *TRIB1* promotes atherosclerosis in mice, which is accompanied by pronounced hyperlipidemia and hepatic and systemic inflammation [82]. However, lack of *TRIB1* in myeloid cells decreases early atheroma formation and reduces atherosclerosis burden in mouse models [83]. A recent meta-analysis reveals that the TRIB1 SNP (Rs2954029) A allele present in the 8q24 locus is positively associated with the risk of coronary artery disease (CAD) combined with stroke [84]. Several studies have also explored the association between TRIB1 and non-alcoholic liver disease and hepatic steatosis in terms of SNPs (rs6982502, rs2954021, rs17321515, rs2954029) in different populations [40,85,86,87]. Transcriptomics studies in similar mouse models reveal genes enriched in VLDL assembly, TG biosynthesis, gluconeogenesis, and glycogen synthesis pathways are upregulated after *TRIB1* overexpression [40,85,86,87]. In order to exploit TRIB1’s ability to modulate lipid metabolism, Nagiec et al. identified a small molecule BRD0418, a benzofuran compound that increases *TRIB1* expression in HepG2 cells, leading to reduced VLDL production, cholesterol biosynthesis, and increased LDL uptake [88]. The cytokine oncostatin M, which is shown to upregulate *LDLR* gene transcription in HepG2 cells [89], also acts by increasing *TRIB1* gene expression [88]. Another tricyclic glycal compound, BRD8518, was later identified to have higher potency than BRD0418 in inducing *TRIB1* and *LDLR* gene expression. This compound affects the MAPK pathway but is not suitable for in vivo testing because of its ADME profile [90]. TRIB1 is also suggested to influence obesity by regulating brown adipose tissue mitochondrial function via respiratory chain complex III [91]. Of note, TRIB1 is shown to negatively regulate B cells in systemic lupus erythematosus via its interaction with CD72, an effector of autoimmunity [48].

## 4. Role of TRIB1 in Cancer Therapy Resistance

Therapy resistance has always been considered the biggest roadblock in cancer treatment and relapse. Based on the above discussion, it is clear that TRIB1 plays an important role in the malignant transformation of various tumors and modulates several mechanisms for tumor sustenance. Several reports have shown that its involvement in various pathways, such as PI3K-AKT, MAPK, p53-HDAC1, and NF-кB, among others, suggests TRIB1 plays a role in cancer therapy resistance. *TRIB1* was identified as an exclusive downstream effector of mutant *PIK3CA* in genetically modified lung epithelial cells [92]. A later report showed that downregulation of p53 by the TRIB1-HDAC1 interaction induces resistance to cisplatin chemotherapy and cancer stem cell enrichment in non-small cell lung carcinoma (NSCLC) [93]. Similarly, the *TRIB1* gene was identified as part of a gene signature induced in breast cancer cell lines in response to long-term paclitaxel treatment, causing treatment resistance [94]. TRIB1 overexpression causes resistance to ATRA (all-trans retinoic acid) treatment during acute promyelocytic leukemia in exclusively sensitive myeloid cells expressing the PML/RARA fusion protein by preventing their differentiation through the downregulation of C/EBPα [95]. We and others have shown that *TRIB1* mRNA and protein levels are upregulated by radiation and temozolomide treatment in glioma cells, causing a decrease in treatment-induced cell death [34,80]. In primary GBM cells, TRIB1 causes upregulation of the ERK and Akt pathways and modulation of p53 function through COP1 and HDAC1, further contributing to therapy resistance [34]. TRIB1 negatively regulates the anti-tumor cytokine IL-15 in TAMs in breast tumors, causing a decrease in T-cells that promote anti-tumor responses [73]. TRIB1 has been identified as a negative regulator of T-cell receptor (TCR) signaling via its interaction with MALT1, a TCR activator, leading to disruption of MALT1 signaling complexes responsible for optimal T cell activation and function [49]. Deletion of TRIB1 from T cells has been reported to improve PD-L1 immune checkpoint blockade, suggesting a role for TRIB1 in immunotherapy resistance [96]. A recent report has similarly shown that in a CRC mouse model, T cell recruitment to the tumor was improved after TRIB3 loss, thereby sensitizing the tumor to immune checkpoint blockade therapy [97]. The roles of TRIB1 in cancer and therapy resistance are further discussed in Table 2.

## 5. Potential Strategies to Target TRIB1

In light of the above discussion, it is clear that TRIB1 is involved in the tumor maintenance and therapy resistance of various cancers. Therefore, inhibiting TRIB1 can be considered a potential strategy for targeted cancer therapy that would alter the function/activity of important proteins such as Akt and MAPK in normal cells. In general, TRIB1 and many other pseudokinases have been considered “undruggable” for a very long time because, unlike conventional kinases, they do not possess an ATP binding domain, which has so far been the most druggable site in these proteins. On the contrary, the pseudokinases that possess weak catalytic activity (such as TRIB2, the JAK family, and HER3) have been targeted by small molecules against their catalytic site and demonstrated inhibition of their pseudokinase activities. There are a variety of FDA-approved JAK inhibitors for different diseases that act not only by targeting the ATP binding domain but also through allosteric inhibition [103]. Ruxolitinib was the first FDA-approved small-molecule inhibitor of JAK1/2 for the treatment of BCR-ABL1-negative myeloproliferative neoplasms and acts through competitive inhibition of the ATP-binding catalytic site on the kinase domain [104]. 

Given the variety of functions performed by TRIB1, several approaches can be utilized to disrupt its efficient operation. The most prominent function of TRIB1 is to act as a scaffold for the sequestration of signaling molecules, for which it adapts “SLE-in” (on) and “SLE-out” (off) conformations that allow for the substrates/effectors to bind. Small-molecule compounds locking TRIB1 in the SLE-in conformation would allow for blocking any substrate from binding, and locking in the SLE-out conformation would maintain TRIB1 in its auto-inhibitory conformation. Jamieson et al. have tested several compounds for their potential to stabilize TRIB1 in either of these conformations and reported moderate success [23]. Foulkes et al. reported that pre-existing EGFR inhibitors can target the low-affinity ATP binding site on TRIB2 and cause either its stabilization or destabilization [105]. Mutant K-Ras has been an “undruggable” target for years in lung cancer treatment, but the design of a covalent inhibitor that locks this protein in its “inactive conformation” has provided great promise in targeting this protein [106]. The recent resolution of the TRIB1 crystal structure and deeper understanding of the mechanisms of TRIB1 action have revealed new substrates and sites that can be used to design small-molecule inhibitors that are not only effective but are also blood–brain-barrier permeable. 

Another potential approach for targeting TRIB1 could be the induction of its degradation through PROTACs (proteolysis targeting chimeras) and HyT (hydrophobic tagging) to reduce its levels inside the cell. PROTACs are bifunctional molecules that cause the degradation of a protein(s) of interest by bringing them within close proximity of an E3 ligase [107]. HyT, on the other hand, tags protein(s) of interest with hydrophobic fragments so that they are recognized as misfolded and damaged proteins and are therefore cleared through protein quality control mechanisms [108]. The pseudokinase Her3 has been targeted using both approaches. PROTAC selectively degrades HER3 but does not abrogate its downstream signaling [109], whereas HyT induces partial HER3 degradation and blocks HER3-dependent signaling in cell line models [110]. PROTAC 23 developed against IRAK3 pseudokinase exhibits degradation of around 98% IRAK3 protein in THP1 cells [111]. MLKL is another pseudokinase that has been successfully targeted using PROTAC 36 in the TSZ model of necroptosis, demonstrating an inhibition of cell death [112]. Other alternative approaches worth exploration include allosteric inhibition, antibodies, proteasome inhibitors, and irreversible inhibitors, among others. 

## 6. Conclusions

TRIB1 plays a variety of roles in both health and disease. Although it may not phosphorylate any downstream substrates like a canonical kinase, it certainly acts as an intermediate for the sequestration of proteins to carry out important physiological functions such as differentiation, lipid metabolism, and innate immune function, among others. As was identified by Sanchez-Vega et al., there are at least ten cell signaling pathways that are found to be altered in different cancers [113]. Of these pathways, TRIB1 has been shown to play a role in the RTK/RAS, PI3K, Myc, p53, and cell cycle pathways in different tumor types, suggesting the importance of this pseudokinase in oncogenic signaling. TRIB1 and pseudokinases in general have only recently gained limelight in the cancer field for providing a new insight into tumorigenic processes. The implication of TRIB1 in malignant transformation also provides an opportunity for drug development for effective therapies. However, the lack of a catalytic domain on this protein may pose some hurdles, but its alternating “active” and “inactive” conformations hold potential as an attractive vulnerability to design effective drugs that would block its scaffolding function and disrupt downstream oncogenic signaling. PROTACs are an up-and-coming approach that have shown success in hitting undruggable targets and therefore warrants further exploration with regards to TRIB1. In conclusion, targeting TRIB1 presents an opportunity for cancer treatment as well as other metabolic disorders in which TRIB1 has been shown to play a role. 

## Figures and Tables

**Table 2:** TRIB1 in cancer and treatment resistance.

Cancer	Biological Significance of TRIB1	References	Cancer Treatment	Role of TRIB1 in Treatment Resistance	References
Acute myeloid leukemia	TRIB1 overexpression drives Hoxa9-induced leukemogenesis by decreasing C/EBPα to induce the enhancer programs at *Erg* and *Spns2* loci. TRIB1 interacts with MEK1 to activate the MAPK pathway and enhances self-renewal of malignant bone marrow cells.	[12,63]	All-trans retinoic acid	TRIB1 overexpression causes resistance to ATRA treatment in acute promyelocytic leukemia through downregulation of C/EBPα.	[95]
			Cytarabine Daunorubicin Idarubicin	None reported	
Prostate cancer	*TRIB1* and *cMYC* are co-amplified in prostate cancer. TRIB1 promotes secretion of CXCL and IL-8 by inhibiting IкB-ζ expression and induces tumor growth. TRIB1 upregulates GRP78 to promote the occurrence and survival of prostate tumor cells. MiR-224, which targets *TRIB1*, is downregulated in prostate cancer.	[70,71,98]	Docetaxel	Increased expression of TRIB1 promotes resistance to docetaxel.	[99]
			Mitoxantrone	Increased *TRIB1* gene expression is associated with resistance to mitoxantrone.	[100]
			Estramustine Carboplatin	None reported	
Breast cancer	*TRIB1* and *cMYC* are co-amplified in breast cancer patients. TRIB1 regulates both G1/S and G2/M transition in breast cancer cells. TRIB1 interacts with β-catenin and its co-factor FERMT2 and may regulate β-catenin activity. TRIB1 also interacts with ER co-factor ZBTB7A and could influence ER-associated transcription. TRIB1 reduces DR5 and TRAIL-induced apoptosis by elevating NF-кB signaling. TRIB1 negatively regulates the anti-tumor cytokine IL-15 in tumor associated macrophages to decrease T-cell infiltration and anti-tumor responses.	[36,46,73]	Paclitaxel	Long-term paclitaxel treatment in breast cancer cell lines leads to induction of *TRIB1* gene and paclitaxel resistance.	[94]
			Eribulin	Inhibition of TRIB1 enhanced the anti-proliferation effect of eribulin in triple-negative breast cancer cells.	[101]
			Doxorubicin Epirubicin 5-fluorouracil (5-FU) Capecitabine Cyclophosphamide Carboplatin Ixabepilone Vinorelbine Gemcitabine	None reported	
Colorectal cancer	*TRIB1* and *cMYC* are co-amplified in cells and in patients. TRIB1 promotes migration and invasion of CRC cells through the activation of FAK/Src and the ERK pathway to upregulate MMP-2 expression.	[74,76]	Irinotecan	Treatment with the active metabolite of irinotecan, SN38, acutely induces *TRIB1* gene expression in colon cancer cells. Increased *TRIB1* gene expression is associated with resistance to irinotecan.	[102]
			5-FU Capecitabine Oxaliplatin Trifluridine and tipiracil	None reported	
Pancreatic cancer	A single nucleotide polymorphism in the *TRIB1* gene (rs2980879) is associated with pancreatic cancer in the Chinese Han population.	[77]	Gemcitabine 5-FU Oxaliplatin Albumin-bound paclitaxel (Abrxane) Capecitabine Cisplatin Irinotecan	None reported	
Multiple myeloma	*TRIB1* gene expression is higher in bone marrow mononuclear cells of multiple myeloma patients with progressive disease compared to those in remission. Patients with progressive disease have increased percentage of M2 macrophages and suggests a role of TRIB1 in M2 macrophage polarization in multiple myeloma potentially via the JAK/STAT pathway.	[78]	Cyclophosphamide Etoposide Doxorubicin Melphalan Bendamustine	None reported	
Hepatocellular carcinoma	*TRIB1* is upregulated in hepatocellular carcinoma cells and tissues. TRIB1 promotes hepatocellular carcinoma tumorigenesis and invasiveness through a feedback loop involving miR-23a and p53.	[79]	Sorafenib Gemcitabine Oxaliplatin Cisplatin Doxorubicin 5-FU Capecitabine Mitoxantrone	None reported	
Glioma	TRIB1 causes upregulation of ERK and Akt pathways. TRIB1 inhibits activity of p53 in glioma through COP1 and HDAC1, leading to treatment resistance.	[34,80]	Radiation	*TRIB1* mRNA and protein levels increase after radiation treatment in glioma cells causing a decrease in treatment-induced cell death.	[34,80]
			Temozolomide	*TRIB1* mRNA and protein levels increase after temozolomide treatment in glioma cells causing a decrease in treatment-induced cell death.	[34,80]
Non-small cell lung cancer	TRIB1 interacts with HDAC1 to deacetylate and inactivate p53. TRIB1 participates in the abnormal activation of the PI3K/Akt pathway through regulation by mutant PIK3CA.	[92,93]	Cisplatin	Cisplatin treatment resulted in C/EBP-*β*-dependent increasing of TRIB1, which forms a complex with HDAC1 to downregulate p53 and induce resistance to cisplatin.	[93]
			Vinorelbine	Cisplatin pre-treatment of lung cancer cells resulted in increased resistance to vinorelbine.	[93]
			Carboplatin Paclitaxel Albumin-bound paclitaxel (Abraxane) Docetaxel Gemcitabine Etoposide Pemetrexed	None reported	

## List of Abbreviations

AA	Amino Acid
AML	Acute Myeloid Leukemia
C/EBPα	CAAT Enhancer Binding Protein α
CAMK	Ca^2+^/Calmodulin-Dependent Protein Kinase
ChREBP	Carbohydrate Responsive Element Binding Protein
COP1	Constitutive Photomorphogenic 1
EMT	Epithelial to Mesenchymal Transition
ER	Estrogen Receptor
EGFR	Epidermal Growth Factor Receptor
Foxp3	Forkhead box p3
HNF4A	Hepatocyte Nuclear Factor 4-Alpha
IRAK3	Interleukin-1 receptor-associated kinase 3
JNK	c-Jun N-terminal Kinase
MLKL	Mixed Lineage Kinase-Like
MLX	Max like protein x
NF-кB	Nuclear factor kappa B
Nrf2	Nuclear Factor Erythroid 2-Related Factor 2
MALT1	Mucosa-Associated Lymphoid Tissue protein 1
PML/RARA	Promyelocytic Leukemia/Retinoic Acid Receptor Alpha
TLR	Toll-like Receptor
HER3	Human Epidermal Growth Factor Receptor 3
TRAIL	TNF-related apoptosis-inducing ligand
VRK3	Vaccinia Related Kinase

## References

[1] Manning G., Whyte D.B., Martinez R., Hunter T., Sudarsanam S. (2002). The protein kinase complement of the human genome. Science.

[2] Shi F., Telesco S.E., Liu Y., Radhakrishnan R., Lemmon M.A. (2010). ErbB3/HER3 intracellular domain is competent to bind ATP and catalyze autophosphorylation. Proc. Natl. Acad. Sci. USA.

[3] Kang T.H., Kim K.T. (2006). Negative regulation of ERK activity by VRK3-mediated activation of VHR phosphatase. Nat. Cell Biol..

[4] Eyers P.A., Keeshan K., Kannan N. (2017). Tribbles in the 21st Century: The Evolving Roles of Tribbles Pseudokinases in Biology and Disease. Trends Cell Biol..

[5] Sun L., Wang H., Wang Z., He S., Chen S., Liao D., Wang L., Yan J., Liu W., Lei X. (2012). Mixed lineage kinase domain-like protein mediates necrosis signaling downstream of RIP3 kinase. Cell.

[6] Kung J.E., Jura N. (2019). Prospects for pharmacological targeting of pseudokinases. Nat. Rev. Drug Discov..

[7] Wilkin F., Savonet V., Radulescu A., Petermans J., Dumont J.E., Maenhaut C. (1996). Identification and characterization of novel genes modulated in the thyroid of dogs treated with methimazole and propylthiouracil. J. Biol. Chem..

[8] Sharova L.V., Sharov A.A., Nedorezov T., Piao Y., Shaik N., Ko M.S. (2009). Database for mRNA half-life of 19 977 genes obtained by DNA microarray analysis of pluripotent and differentiating mouse embryonic stem cells. DNA Res..

[9] Soubeyrand S., Martinuk A., Lau P., McPherson R. (2016). TRIB1 Is Regulated Post-Transcriptionally by Proteasomal and Non-Proteasomal Pathways. PLoS ONE.

[10] Ruiz-Cantos M., Hutchison C.E., Shoulders C.C. (2021). Musings from the Tribbles Research and Innovation Network. Cancers.

[11] Fagerberg L., Hallström B.M., Oksvold P., Kampf C., Djureinovic D., Odeberg J., Habuka M., Tahmasebpoor S., Danielsson A., Edlund K. (2014). Analysis of the human tissue-specific expression by genome-wide integration of transcriptomics and antibody-based proteomics. Mol. Cell Proteom..

[12] Yokoyama T., Kanno Y., Yamazaki Y., Takahara T., Miyata S., Nakamura T. (2010). Trib1 links the MEK1/ERK pathway in myeloid leukemogenesis. Blood.

[13] Kung J.E., Jura N. (2019). The pseudokinase TRIB1 toggles an intramolecular switch to regulate COP1 nuclear export. EMBO J..

[14] Niespolo C., Johnston J.M., Deshmukh S.R., Satam S., Shologu Z., Villacanas O., Sudbery I.M., Wilson H.L., Kiss-Toth E. (2020). Tribbles-1 Expression and Its Function to Control Inflammatory Cytokines, Including Interleukin-8 Levels are Regulated by miRNAs in Macrophages and Prostate Cancer Cells. Front. Immunol..

[15] Soubeyrand S., Lau P., McPherson R. (2023). Regulation of TRIB1 abundance in hepatocyte models in response to proteasome inhibition. Sci. Rep..

[16] Mata J., Curado S., Ephrussi A., Rørth P. (2000). Tribbles coordinates mitosis and morphogenesis in Drosophila by regulating string/CDC25 proteolysis. Cell.

[17] Grosshans J., Wieschaus E. (2000). A genetic link between morphogenesis and cell division during formation of the ventral furrow in Drosophila. Cell.

[18] Seher T.C., Leptin M. (2000). Tribbles, a cell-cycle brake that coordinates proliferation and morphogenesis during Drosophila gastrulation. Curr. Biol..

[19] Kiss-Toth E., Wyllie D.H., Holland K., Marsden L., Jozsa V., Oxley K.M., Polgar T., Qwarnstrom E.E., Dower S.K. (2006). Functional mapping and identification of novel regulators for the Toll/Interleukin-1 signalling network by transcription expression cloning. Cell Signal.

[20] Johnston J., Kiss-Toth E. (2016). TRIB1 (tribbles pseudokinase 1). Atlas Genet. Cytogenet. Oncol. Haematol..

[21] Foulkes D.M., Byrne D.P., Bailey F.P., Eyers P.A. (2015). Tribbles pseudokinases: Novel targets for chemical biology and drug discovery?. Biochem. Soc. Trans..

[22] Murphy J.M., Nakatani Y., Jamieson S.A., Dai W., Lucet I.S., Mace P.D. (2015). Molecular Mechanism of CCAAT-Enhancer Binding Protein Recruitment by the TRIB1 Pseudokinase. Structure.

[23] Jamieson S.A., Ruan Z., Burgess A.E., Curry J.R., McMillan H.D., Brewster J.L., Dunbier A.K., Axtman A.D., Kannan N., Mace P.D. (2018). Substrate binding allosterically relieves autoinhibition of the pseudokinase TRIB1. Sci. Signal..

[24] Uljon S., Xu X., Durzynska I., Stein S., Adelmant G., Marto J.A., Pear W.S., Blacklow S.C. (2016). Structural Basis for Substrate Selectivity of the E3 Ligase COP1. Structure.

[25] Goldberg J., Nairn A.C., Kuriyan J. (1996). Structural basis for the autoinhibition of calcium/calmodulin-dependent protein kinase I. Cell.

[26] Eyers P.A. (2015). TRIBBLES: A Twist in the Pseudokinase Tail. Structure.

[27] Yoshida A., Kato J.Y., Nakamae I., Yoneda-Kato N. (2013). COP1 targets C/EBPα for degradation and induces acute myeloid leukemia via Trib1. Blood.

[28] Richmond L., Keeshan K. (2020). Pseudokinases: A tribble-edged sword. FEBS J..

[29] Rørth P., Szabo K., Texido G. (2000). The level of C/EBP protein is critical for cell migration during Drosophila oogenesis and is tightly controlled by regulated degradation. Mol. Cell.

[30] Radomska H.S., Huettner C.S., Zhang P., Cheng T., Scadden D.T., Tenen D.G. (1998). CCAAT/enhancer binding protein alpha is a regulatory switch sufficient for induction of granulocytic development from bipotential myeloid progenitors. Mol. Cell Biol..

[31] Satoh T., Kidoya H., Naito H., Yamamoto M., Takemura N., Nakagawa K., Yoshioka Y., Morii E., Takakura N., Takeuchi O. (2013). Critical role of Trib1 in differentiation of tissue-resident M2-like macrophages. Nature.

[32] Imajo M., Nishida E. (2010). Human Tribbles homolog 1 functions as a negative regulator of retinoic acid receptor. Genes Cells.

[33] Das R., Sebo Z., Pence L., Dobens L.L. (2014). Drosophila tribbles antagonizes insulin signaling-mediated growth and metabolism via interactions with Akt kinase. PLoS ONE.

[34] Singh K., Han C., Fleming J.L., Becker A.P., McElroy J., Cui T., Johnson B., Kumar A., Sebastian E., Showalter C.A. (2023). TRIB1 confers therapeutic resistance in GBM cells by activating the ERK and Akt pathways. Sci. Rep..

[35] Yu J.M., Sun W., Wang Z.H., Liang X., Hua F., Li K., Lv X.X., Zhang X.W., Liu Y.Y., Yu J.J. (2019). TRIB3 supports breast cancer stemness by suppressing FOXO1 degradation and enhancing SOX2 transcription. Nat. Commun..

[36] Gendelman R., Xing H., Mirzoeva O.K., Sarde P., Curtis C., Feiler H.S., McDonagh P., Gray J.W., Khalil I., Korn W.M. (2017). Bayesian Network Inference Modeling Identifies TRIB1 as a Novel Regulator of Cell-Cycle Progression and Survival in Cancer Cells. Cancer Res..

[37] Kiss-Toth E., Bagstaff S.M., Sung H.Y., Jozsa V., Dempsey C., Caunt J.C., Oxley K.M., Wyllie D.H., Polgar T., Harte M. (2004). Human tribbles, a protein family controlling mitogen-activated protein kinase cascades. J. Biol. Chem..

[38] Sung H.Y., Guan H., Czibula A., King A.R., Eder K., Heath E., Suvarna S.K., Dower S.K., Wilson A.G., Francis S.E. (2007). Human tribbles-1 controls proliferation and chemotaxis of smooth muscle cells via MAPK signaling pathways. J. Biol. Chem..

[39] Arndt L., Dokas J., Gericke M., Kutzner C.E., Müller S., Jeromin F., Thiery J., Burkhardt R. (2018). Tribbles homolog 1 deficiency modulates function and polarization of murine bone marrow-derived macrophages. J. Biol. Chem..

[40] Ishizuka Y., Nakayama K., Ogawa A., Makishima S., Boonvisut S., Hirao A., Iwasaki Y., Yada T., Yanagisawa Y., Miyashita H. (2014). TRIB1 downregulates hepatic lipogenesis and glycogenesis via multiple molecular interactions. J. Mol. Endocrinol..

[41] Soubeyrand S., Martinuk A., McPherson R. (2017). TRIB1 is a positive regulator of hepatocyte nuclear factor 4-alpha. Sci. Rep..

[42] Makishima S., Boonvisut S., Ishizuka Y., Watanabe K., Nakayama K., Iwamoto S. (2015). Sin3A-associated protein, 18 kDa, a novel binding partner of TRIB1, regulates MTTP expression. J. Lipid Res..

[43] Sun X., Wang S., Miao X., Zeng S., Guo Y., Zhou A., Chen Y., Lv F., Fan Z., Wang Y. (2023). TRIB1 regulates liver regeneration by antagonizing the NRF2-mediated antioxidant response. Cell Death Dis..

[44] Ostertag A., Jones A., Rose A.J., Liebert M., Kleinsorg S., Reimann A., Vegiopoulos A., Berriel Diaz M., Strzoda D., Yamamoto M. (2010). Control of adipose tissue inflammation through TRB1. Diabetes.

[45] Dugast E., Kiss-Toth E., Docherty L., Danger R., Chesneau M., Pichard V., Judor J.P., Pettré S., Conchon S., Soulillou J.P. (2013). Identification of tribbles-1 as a novel binding partner of Foxp3 in regulatory T cells. J. Biol. Chem..

[46] McMillan H.D., Papachristou E.K., Hazlett J., Omarjee S., Carroll J.S., Black M.A., Mace P.D., Dunbier A.K. (2023). TRIB1 modulates transcriptional programming in breast cancer cells to regulate cell proliferation. bioRxiv.

[47] Miyajima C., Inoue Y., Hayashi H. (2015). Pseudokinase tribbles 1 (TRB1) negatively regulates tumor-suppressor activity of p53 through p53 deacetylation. Biol. Pharm. Bull..

[48] Simoni L., Delgado V., Ruer-Laventie J., Bouis D., Soley A., Heyer V., Robert I., Gies V., Martin T., Korganow A.S. (2018). Trib1 Is Overexpressed in Systemic Lupus Erythematosus, While It Regulates Immunoglobulin Production in Murine B Cells. Front. Immunol..

[49] Rome K.S., Stein S.J., Kurachi M., Petrovic J., Schwartz G.W., Mack E.A., Uljon S., Wu W.W., DeHart A.G., McClory S.E. (2020). Trib1 regulates T cell differentiation during chronic infection by restraining the effector program. J. Exp. Med..

[50] Kathiresan S., Melander O., Guiducci C., Surti A., Burtt N.P., Rieder M.J., Cooper G.M., Roos C., Voight B.F., Havulinna A.S. (2008). Six new loci associated with blood low-density lipoprotein cholesterol, high-density lipoprotein cholesterol or triglycerides in humans. Nat. Genet..

[51] Burkhardt R., Toh S.A., Lagor W.R., Birkeland A., Levin M., Li X., Robblee M., Fedorov V.D., Yamamoto M., Satoh T. (2010). Trib1 is a lipid- and myocardial infarction-associated gene that regulates hepatic lipogenesis and VLDL production in mice. J. Clin. Investig..

[52] Bauer R.C., Sasaki M., Cohen D.M., Cui J., Smith M.A., Yenilmez B.O., Steger D.J., Rader D.J. (2015). Tribbles-1 regulates hepatic lipogenesis through posttranscriptional regulation of C/EBPα. J. Clin. Investig..

[53] Quiroz-Figueroa K., Vitali C., Conlon D.M., Millar J.S., Tobias J.W., Bauer R.C., Hand N.J., Rader D.J. (2021). TRIB1 regulates LDL metabolism through CEBPα-mediated effects on the LDL receptor in hepatocytes. J. Clin. Investig..

[54] Ha E.E., Quartuccia G.I., Ling R., Xue C., Karikari R.A., Hernandez-Ono A., Hu K.Y., Matias C.V., Imam R., Cui J. (2022). Adipocyte-specific tribbles pseudokinase 1 regulates plasma adiponectin and plasma lipids in mice. Mol. Metab..

[55] Chambers J.C., Zhang W., Sehmi J., Li X., Wass M.N., Van der Harst P., Holm H., Sanna S., Kavousi M., Baumeister S.E. (2011). Genome-wide association study identifies loci influencing concentrations of liver enzymes in plasma. Nat. Genet..

[56] Bhairavabhotla R., Kim Y.C., Glass D.D., Escobar T.M., Patel M.C., Zahr R., Nguyen C.K., Kilaru G.K., Muljo S.A., Shevach E.M. (2016). Transcriptome profiling of human FoxP3+ regulatory T cells. Hum. Immunol..

[57] Miyajima C., Itoh Y., Inoue Y., Hayashi H. (2015). Positive Regulation of Interleukin-2 Expression by a Pseudokinase, Tribbles 1, in Activated T Cells. Biol. Pharm. Bull..

[58] Yamamoto M., Uematsu S., Okamoto T., Matsuura Y., Sato S., Kumar H., Satoh T., Saitoh T., Takeda K., Ishii K.J. (2007). Enhanced TLR-mediated NF-IL6 dependent gene expression by Trib1 deficiency. J. Exp. Med..

[59] Liu Y.H., Tan K.A., Morrison I.W., Lamb J.R., Argyle D.J. (2013). Macrophage migration is controlled by Tribbles 1 through the interaction between C/EBPβ and TNF-α. Vet. Immunol. Immunopathol..

[60] Ashton-Chess J., Giral M., Mengel M., Renaudin K., Foucher Y., Gwinner W., Braud C., Dugast E., Quillard T., Thebault P. (2008). Tribbles-1 as a novel biomarker of chronic antibody-mediated rejection. J. Am. Soc. Nephrol..

[61] Röthlisberger B., Heizmann M., Bargetzi M.J., Huber A.R. (2007). TRIB1 overexpression in acute myeloid leukemia. Cancer Genet. Cytogenet..

[62] Döhner H. (2007). Implication of the molecular characterization of acute myeloid leukemia. Hematol. Am. Soc. Hematol. Educ. Program..

[63] Yoshino S., Yokoyama T., Sunami Y., Takahara T., Nakamura A., Yamazaki Y., Tsutsumi S., Aburatani H., Nakamura T. (2021). Trib1 promotes acute myeloid leukemia progression by modulating the transcriptional programs of Hoxa9. Blood.

[64] Pineault N., Helgason C.D., Lawrence H.J., Humphries R.K. (2002). Differential expression of Hox, Meis1, and Pbx1 genes in primitive cells throughout murine hematopoietic ontogeny. Exp. Hematol..

[65] Jin G., Yamazaki Y., Takuwa M., Takahara T., Kaneko K., Kuwata T., Miyata S., Nakamura T. (2007). Trib1 and Evi1 cooperate with Hoxa and Meis1 in myeloid leukemogenesis. Blood.

[66] Kroon E., Krosl J., Thorsteinsdottir U., Baban S., Buchberg A.M., Sauvageau G. (1998). Hoxa9 transforms primary bone marrow cells through specific collaboration with Meis1a but not Pbx1b. EMBO J..

[67] Lawrence H.J., Rozenfeld S., Cruz C., Matsukuma K., Kwong A., Kömüves L., Buchberg A.M., Largman C. (1999). Frequent co-expression of the HOXA9 and MEIS1 homeobox genes in human myeloid leukemias. Leukemia.

[68] Mohr S., Doebele C., Comoglio F., Berg T., Beck J., Bohnenberger H., Alexe G., Corso J., Ströbel P., Wachter A. (2017). Hoxa9 and Meis1 Cooperatively Induce Addiction to Syk Signaling by Suppressing miR-146a in Acute Myeloid Leukemia. Cancer Cell.

[69] Shahrouzi P., Astobiza I., Cortazar A.R., Torrano V., Macchia A., Flores J.M., Niespolo C., Mendizabal I., Caloto R., Ercilla A. (2020). Genomic and Functional Regulation of TRIB1 Contributes to Prostate Cancer Pathogenesis. Cancers.

[70] Mashima T., Soma-Nagae T., Migita T., Kinoshita R., Iwamoto A., Yuasa T., Yonese J., Ishikawa Y., Seimiya H. (2014). TRIB1 supports prostate tumorigenesis and tumor-propagating cell survival by regulation of endoplasmic reticulum chaperone expression. Cancer Res..

[71] Liu Z.Z., Han Z.D., Liang Y.K., Chen J.X., Wan S., Zhuo Y.J., Cai Z.D., Deng Y.L., Lin Z.Y., Mo R.J. (2019). TRIB1 induces macrophages to M2 phenotype by inhibiting IKB-zeta in prostate cancer. Cell Signal.

[72] Papachristou E.K., Kishore K., Holding A.N., Harvey K., Roumeliotis T.I., Chilamakuri C.S.R., Omarjee S., Chia K.M., Swarbrick A., Lim E. (2018). A quantitative mass spectrometry-based approach to monitor the dynamics of endogenous chromatin-associated protein complexes. Nat. Commun..

[73] Kim T., Johnston J., Castillo-Lluva S., Cimas F.J., Hamby S., Gonzalez-Moreno S., Villarejo-Campos P., Goodall A.H., Velasco G., Ocana A. (2022). TRIB1 regulates tumor growth via controlling tumor-associated macrophage phenotypes and is associated with breast cancer survival and treatment response. Theranostics.

[74] Camps J., Nguyen Q.T., Padilla-Nash H.M., Knutsen T., McNeil N.E., Wangsa D., Hummon A.B., Grade M., Ried T., Difilippantonio M.J. (2009). Integrative genomics reveals mechanisms of copy number alterations responsible for transcriptional deregulation in colorectal cancer. Genes. Chromosomes Cancer.

[75] Briffa R., Um I., Faratian D., Zhou Y., Turnbull A.K., Langdon S.P., Harrison D.J. (2015). Multi-Scale Genomic, Transcriptomic and Proteomic Analysis of Colorectal Cancer Cell Lines to Identify Novel Biomarkers. PLoS ONE.

[76] Wang Y., Wu N., Pang B., Tong D., Sun D., Sun H., Zhang C., Sun W., Meng X., Bai J. (2017). TRIB1 promotes colorectal cancer cell migration and invasion through activation MMP-2 via FAK/Src and ERK pathways. Oncotarget.

[77] Lu X.X., Hu J.J., Fang Y., Wang Z.T., Xie J.J., Zhan Q., Deng X.X., Chen H., Jin J.B., Peng C.H. (2014). A case-control study indicates that the TRIB1 gene is associated with pancreatic cancer. Genet. Mol. Res..

[78] Chen H., Li M., Sanchez E., Soof C.M., Bujarski S., Ng N., Cao J., Hekmati T., Zahab B., Nosrati J.D. (2020). JAK1/2 pathway inhibition suppresses M2 polarization and overcomes resistance of myeloma to lenalidomide by reducing TRIB1, MUC1, CD44, CXCL12, and CXCR4 expression. Br. J. Haematol..

[79] Ye Y., Wang G., Zhuang J., He S., Song Y., Ni J., Xia W., Wang J. (2017). The Oncogenic Role of Tribbles 1 in Hepatocellular Carcinoma Is Mediated by a Feedback Loop Involving microRNA-23a and p53. Front. Physiol..

[80] Tang B., Wu W., Zhang Q., Sun Y., Cui Y., Wu F., Wei X., Qi G., Liang X., Tang F. (2015). Inhibition of tribbles protein-1 attenuates radioresistance in human glioma cells. Sci. Rep..

[81] Bauer R.C., Yenilmez B.O., Rader D.J. (2015). Tribbles-1: A novel regulator of hepatic lipid metabolism in humans. Biochem. Soc. Trans..

[82] Arndt L., Hernandez-Resendiz I., Moos D., Dokas J., Müller S., Jeromin F., Wagner R., Ceglarek U., Heid I.M., Höring M. (2023). Deficiency Promotes Hyperlipidemia, Inflammation, and Atherosclerosis in LDL Receptor Knockout Mice. Arterioscler. Thromb. Vasc. Biol..

[83] Johnston J.M., Angyal A., Bauer R.C., Hamby S., Suvarna S.K., Baidžajevas K., Hegedus Z., Dear T.N., Turner M., Wilson H.L. (2019). Myeloid Tribbles 1 induces early atherosclerosis via enhanced foam cell expansion. Sci. Adv..

[84] Jiang J., Chen X., Li C., Du X., Zhou H. (2023). Polymorphisms of TRIB1 genes for coronary artery disease and stroke risk: A systematic review and meta-analysis. Gene.

[85] Kitamoto A., Kitamoto T., Nakamura T., Ogawa Y., Yoneda M., Hyogo H., Ochi H., Mizusawa S., Ueno T., Nakao K. (2014). Association of polymorphisms in GCKR and TRIB1 with nonalcoholic fatty liver disease and metabolic syndrome traits. Endocr. J..

[86] Liu Q., Xue F., Meng J., Liu S.S., Chen L.Z., Gao H., Geng N., Jin W.W., Xin Y.N., Xuan S.Y. (2019). TRIB1 rs17321515 and rs2954029 gene polymorphisms increase the risk of non-alcoholic fatty liver disease in Chinese Han population. Lipids Health Dis..

[87] Soubeyrand S., Martinuk A., Naing T., Lau P., McPherson R. (2016). Role of Tribbles Pseudokinase 1 (TRIB1) in human hepatocyte metabolism. Biochim. Biophys. Acta.

[88] Nagiec M.M., Skepner A.P., Negri J., Eichhorn M., Kuperwasser N., Comer E., Muncipinto G., Subramanian A., Clish C., Musunuru K. (2015). Modulators of hepatic lipoprotein metabolism identified in a search for small-molecule inducers of tribbles pseudokinase 1 expression. PLoS ONE.

[89] Cao A., Wu M., Li H., Liu J. (2011). Janus kinase activation by cytokine oncostatin M decreases PCSK9 expression in liver cells. J. Lipid Res..

[90] Nagiec M.M., Duvall J.R., Skepner A.P., Howe E.A., Bastien J., Comer E., Marie J.C., Johnston S.E., Negri J., Eichhorn M. (2018). Novel tricyclic glycal-based. MedChemComm.

[91] Zhang X., Zhang B., Zhang C., Sun G., Sun X. (2021). Trib1 deficiency causes brown adipose respiratory chain depletion and mitochondrial disorder. Cell Death Dis..

[92] De Marco C., Laudanna C., Rinaldo N., Oliveira D.M., Ravo M., Weisz A., Ceccarelli M., Caira E., Rizzuto A., Zoppoli P. (2017). Specific gene expression signatures induced by the multiple oncogenic alterations that occur within the PTEN/PI3K/AKT pathway in lung cancer. PLoS ONE.

[93] Wang L., Liu X., Ren Y., Zhang J., Chen J., Zhou W., Guo W., Wang X., Chen H., Li M. (2017). Cisplatin-enriching cancer stem cells confer multidrug resistance in non-small cell lung cancer via enhancing TRIB1/HDAC activity. Cell Death Dis..

[94] Jurj A., Pop L.A., Zanoaga O., Ciocan-Cârtiţă C.A., Cojocneanu R., Moldovan C., Raduly L., Pop-Bica C., Trif M., Irimie A. (2020). New Insights in Gene Expression Alteration as Effect of Paclitaxel Drug Resistance in Triple Negative Breast Cancer Cells. Cell Physiol. Biochem..

[95] Keeshan K., Vieugué P., Chaudhury S., Rishi L., Gaillard C., Liang L., Garcia E., Nakamura T., Omidvar N., Kogan S.C. (2016). Co-operative leukemogenesis in acute myeloid leukemia and acute promyelocytic leukemia reveals C/EBPα as a common target of TRIB1 and PML/RARA. Haematologica.

[96] McClory S.E., Bardhan O., Rome K.S., Giles J.R., Baxter A.E., Xu L., Gimotty P.A., Faryabi R.B., Wherry E.J., Pear W.S. (2023). The pseudokinase Trib1 regulates the transition of exhausted T cells to a KLR. Cell Rep..

[97] Shang S., Yang Y.W., Chen F., Yu L., Shen S.H., Li K., Cui B., Lv X.X., Zhang C., Yang C. (2022). TRIB3 reduces CD8. Sci. Transl. Med..

[98] Lin Z.Y., Huang Y.Q., Zhang Y.Q., Han Z.D., He H.C., Ling X.H., Fu X., Dai Q.S., Cai C., Chen J.H. (2014). MicroRNA-224 inhibits progression of human prostate cancer by downregulating TRIB1. Int. J. Cancer.

[99] Tan X., Song X., Fan B., Li M., Zhang A., Pei L. (2022). Exosomal circRNA Scm-like with four malignant brain tumor domains 2 (circ-SFMBT2) enhances the docetaxel resistance of prostate cancer via the microRNA-136-5p/tribbles homolog 1 pathway. Anticancer Drugs.

[100] Györffy B., Surowiak P., Kiesslich O., Denkert C., Schäfer R., Dietel M., Lage H. (2006). Gene expression profiling of 30 cancer cell lines predicts resistance towards 11 anticancer drugs at clinically achieved concentrations. Int. J. Cancer.

[101] Xie X., Lee J., Fuson J.A., Liu H., Iwase T., Yun K., Margain C., Tripathy D., Ueno N.T. (2023). Identification of Kinase Targets for Enhancing the Antitumor Activity of Eribulin in Triple-Negative Breast Cell Lines. Biomedicines.

[102] Allen W.L., Coyle V.M., Jithesh P.V., Proutski I., Stevenson L., Fenning C., Longley D.B., Wilson R.H., Gordon M., Lenz H.J. (2008). Clinical determinants of response to irinotecan-based therapy derived from cell line models. Clin. Cancer Res..

[103] Shawky A.M., Almalki F.A., Abdalla A.N., Abdelazeem A.H., Gouda A.M. (2022). A Comprehensive Overview of Globally Approved JAK Inhibitors. Pharmaceutics.

[104] Mascarenhas J., Hoffman R. (2012). Ruxolitinib: The first FDA approved therapy for the treatment of myelofibrosis. Clin. Cancer Res..

[105] Foulkes D.M., Byrne D.P., Yeung W., Shrestha S., Bailey F.P., Ferries S., Eyers C.E., Keeshan K., Wells C., Drewry D.H. (2018). Covalent inhibitors of EGFR family protein kinases induce degradation of human Tribbles 2 (TRIB2) pseudokinase in cancer cells. Sci. Signal..

[106] Lim S.M., Westover K.D., Ficarro S.B., Harrison R.A., Choi H.G., Pacold M.E., Carrasco M., Hunter J., Kim N.D., Xie T. (2014). Therapeutic targeting of oncogenic K-Ras by a covalent catalytic site inhibitor. Angew. Chem. Int. Ed. Engl..

[107] Lai A.C., Crews C.M. (2017). Induced protein degradation: An emerging drug discovery paradigm. Nat. Rev. Drug Discov..

[108] Neklesa T.K., Crews C.M. (2012). Chemical biology: Greasy tags for protein removal. Nature.

[109] Alabi S.B. (2021). Targeting Oncogenic Kinases and Pseudokinases with Proteolysis Targeting Chimeras.

[110] Xie T., Lim S.M., Westover K.D., Dodge M.E., Ercan D., Ficarro S.B., Udayakumar D., Gurbani D., Tae H.S., Riddle S.M. (2014). Pharmacological targeting of the pseudokinase Her3. Nat. Chem. Biol..

[111] Degorce S.L., Tavana O., Banks E., Crafter C., Gingipalli L., Kouvchinov D., Mao Y., Pachl F., Solanki A., Valge-Archer V. (2020). Discovery of Proteolysis-Targeting Chimera Molecules that Selectively Degrade the IRAK3 Pseudokinase. J. Med. Chem..

[112] Rathje O.H., Perryman L., Payne R.J., Hamprecht D.W. (2023). PROTACs Targeting MLKL Protect Cells from Necroptosis. J. Med. Chem..

[113] Sanchez-Vega F., Mina M., Armenia J., Chatila W.K., Luna A., La K.C., Dimitriadoy S., Liu D.L., Kantheti H.S., Saghafinia S. (2018). Oncogenic Signaling Pathways in The Cancer Genome Atlas. Cell.

